# Delivery of nanovaccine towards lymphoid organs: recent strategies in enhancing cancer immunotherapy

**DOI:** 10.1186/s12951-021-01146-2

**Published:** 2021-11-25

**Authors:** Ting Cai, Huina Liu, Shun Zhang, Jing Hu, Lingxiao Zhang

**Affiliations:** 1 Ningbo Clinical Research Center for Digestive System Tumors, Ningbo Hwa Mei Hospital, University of Chinese Academy of Sciences, Ningbo, 315010 China; 2 Key Laboratory of Diagnosis and Treatment of Digestive System Tumors of Zhejiang Province, Ningbo Hwa Mei Hospital, University of Chinese Academy of Sciences, Ningbo, 315010 China; 3 Ningbo Institute of Life and Health Industry, University of Chinese Academy of Sciences, Ningbo, 315010 China; 4 Key Laboratory of Biomass Chemical Engineering of Ministry of Education, College of Chemical and Biological Engineering, Zhejiang University, Hangzhou, 310027 China; 5 Hangzhou Global Scientific and Technological Innovation Center, Zhejiang University, Hangzhou, 211200 China; 6 College of Pharmaceutical Sciences, Zhejiang University, Hangzhou, 310058 China

**Keywords:** Nanovaccine, Cancer immunotherapy, Lymphoid organ, Vaccine delivery, Immune barrier

## Abstract

With the in-depth exploration on cancer therapeutic nanovaccines, increasing evidence shows that the poor delivery of nanovaccines to lymphoid organs has become the culprit limiting the rapid induction of anti-tumor immune response. Unlike the conventional prophylactic vaccines that mainly form a depot at the injection site to gradually trigger durable immune response, the rapid proliferation of tumors requires an efficient delivery of nanovaccines to lymphoid organs for rapid induction of anti-tumor immunity. Optimization of the physicochemical properties of nanovaccine (*e.g.*, size, shape, charge, colloidal stability and surface ligands) is an effective strategy to enhance their accumulation in lymphoid organs, and nanovaccines with dynamic structures are also designed for precise targeted delivery of lymphoid organs or their subregions. The recent progress of these nanovaccine delivery strategies is highlighted in this review, and the challenges and future direction are also discussed.

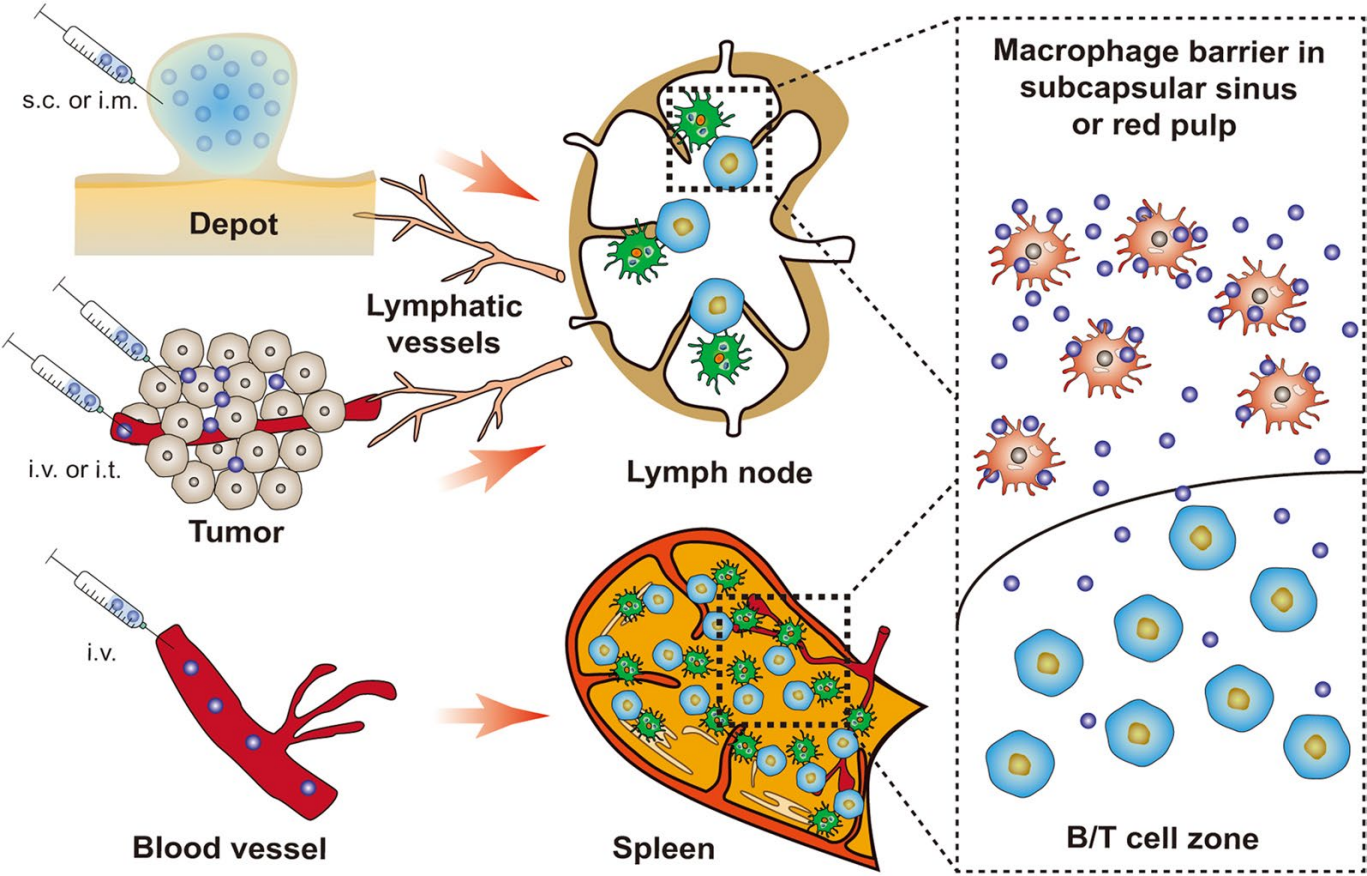

## Introduction

Cancer therapeutic vaccines are important tools against malignant tumors by providing potent immune attack and continuous immune surveillance [1, 2]. Adjuvant as the critical composition of cancer therapeutic vaccine [3], responses for delivering tumor-associated antigens (TAAs) to lymphoid organs and help activate antigen-presenting cells (APCs) [4]. However, the delivery efficiency of clinical adjuvants (such as alum or emulsions) is insufficient, which results in slow induction of anti-tumor immunity and poor cancer immunotherapy efficacy [5]. The potential reason may be due to the formation of vaccine depot at the injection site, which induces anti-tumor immune responses through a time–cost APCs recruitment and antigen delivery process (defined as depot effect) [6–8]. During this process, it takes around 3–7 days for vaccines to reach the highest accumulation in lymph node (LN), a period of time allows the tumor volume to increase by 8–10 times in mice [9]. Therefore, the imbalance between the slow immune induction and the rapid tumor proliferation is a key to limiting the efficacy of cancer immunotherapy.

Lymphoid organs such as LN and spleen with high-intensity of immune cells are major places for vaccines to induce adaptive immune response [10]. In fact, APCs internalized with vaccines can quickly activate T and B cells in lymphoid organs, however most of the vaccines cannot reach APCs in lymphoid organs, which is the main rate-limiting step for immune induction [11, 12]. In order to overcome the shortages of conventional adjuvants, nanoadjuvants with controllable physicochemical properties (*i.e.*, size, surface charge, hydrophobicity, targeting ligand) have been widely investigated [13–18], showing great potential to enhance cancer immunotherapy [19–22]. By enhancing the dynamic interaction with lymphoid organ, more nanovaccines have been successfully delivered to lymphoid organs. For instance, optimization of the nanovaccine softness, surface chemistry, dispersity and size, enables more nanovaccines to actively filtrate into the lymphatic vessels [23, 24] or be captured by spleen (the largest secondary lymphoid organ) [25]. Therefore, this review will first conclude the approaches for nanovaccines to reach lymphoid organs. Then, according to the targeted lymphoid organs, the recently developed vaccine delivery strategies that can greatly enhance the therapeutic efficacy of cancer nanovaccines will be summarized. The design consideration and underlying mechanism will be elucidated, and the concerns of the consensus and currently unresolved issues will also be discussed.

## Approaches to the lymphoid organs

**Fig. 1:**
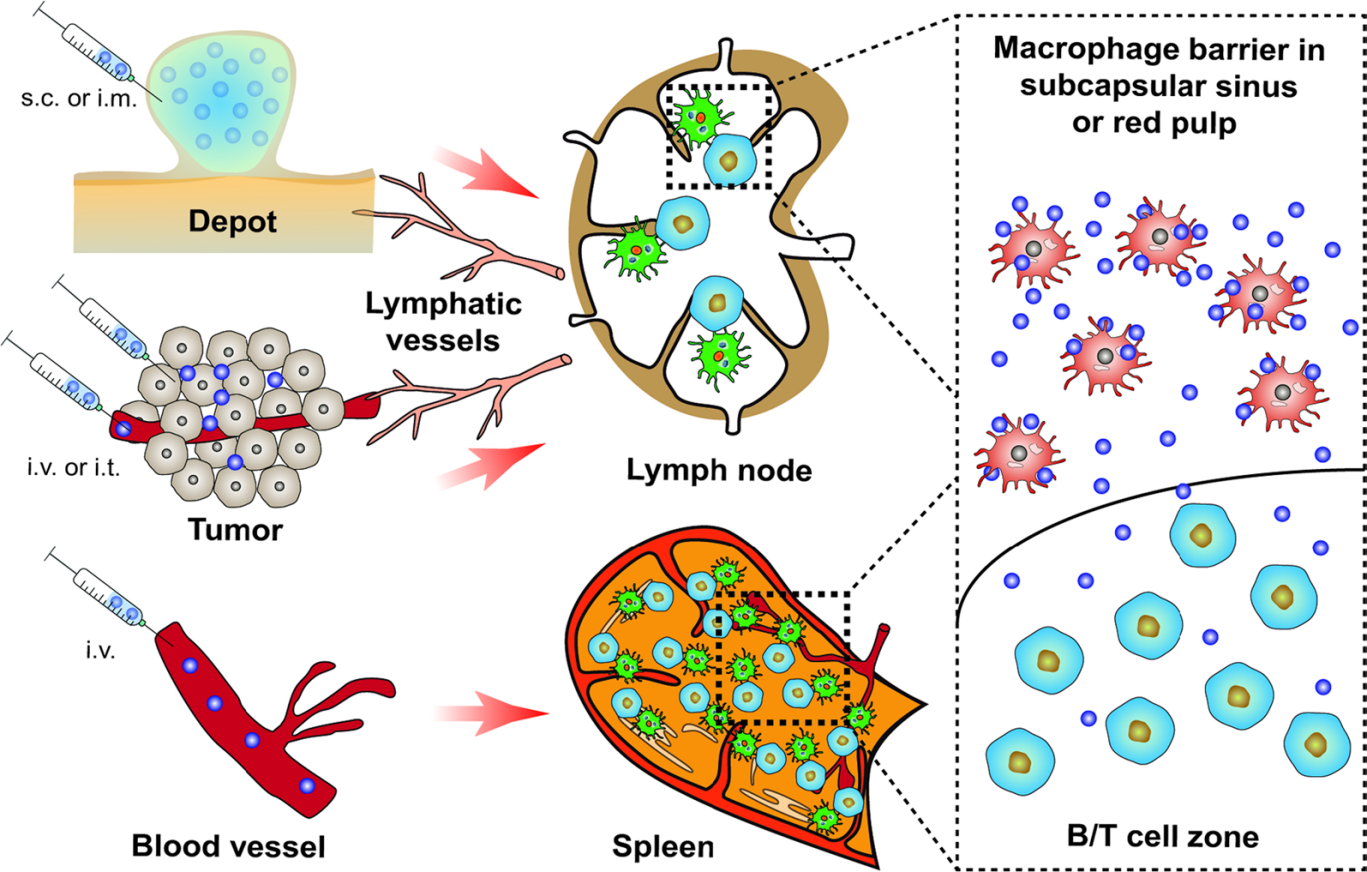
Schematic illustration of the pathways to deliver nanovaccines to lymphoid organs

As shown in Fig. 1, the effective transport of nanovaccines from the injection sites to LNs or the spleen is an important basis for the induction of cancer immunotherapy. Typically, nanovaccines are administered by subcutaneous (s.c.) or intramuscular (i.m.) routes, and subsequently form vaccine depots at the injection site. Ideally, nanovaccines can actively migrate to LNs through lymphatic vessels. More commonly, nanovaccines must rely on the transportation of APCs to achieve enrichment in LNs. For instance, a recent study showed that poly(lactic-co-glycolic acid) (PLGA) nanovaccines with a size of 83 nm could efficiently migrate and deliver antigens from the injection site to LNs. With the particle size increased to 103 nm and 122 nm, the LNs accumulation efficiency of nanovaccines sharply dropped as the larger nanoparticles (NPs) failed to penetrate into the lymphatic vessels [26]. When the nanovaccine reside at the injection site, the shape and size of the particle also determine the microstructure of the vaccine depot, which directly affects the recruitment of APCs and antigen presentation. Kim et al*.* showed that rod-like mesoporous silica nanovaccines with a higher aspect ratio formed a looser vaccine depot, thereby recruiting more APCs to the injection site and inducing stronger immune responses [27]. In addition to the above routes, the functionalized nanoadjuvants injected through the peritumoral, intratumoral (i.t.) or intravenous (i.v.) routes can capture the personalized tumor antigens in situ and present them to the tumor-resident APCs or deliver them to the tumor-associated draining lymph nodes (tdLNs) [28]. In general, these functionalized nanoadjuvants are integrated with chemotherapy drugs, photothermal/photosensitizers or nucleus, which enable them to destroy tumor tissues through chemo-, photothermal/photodynamic or radio-therapy. Then, the released tumor antigens are further captured by the nanoadjuvants and delivered to tdLNs to trigger personalized anti-tumor immunity [29]. In addition, the spleen, the largest secondary lymphatic organ, has also attracted attention for its rapid induction of potent anti-tumor immunity [30]. To achieve efficient spleen accumulation, the size of nanovaccines has been optimized and surface ligands such as albumin- or red blood cell (RBC) membrane have been used to enhance their circulation and spleen-targeted delivery efficiency post i.v. administration [25, 31]. It is worth noting that the macrophage barrier located in the subcapsular sinus of the LN or the red pulp of the spleen is an obstacle preventing the nanovaccine from reaching T or B lymphocytes [32]. To overcome this, strategies have been spawned to help nanovaccine bypass the macrophage barrier and interact with B/T cell zones [33–35]. This review will summarize the recent cancer nanovaccine delivery strategies toward lymphoid organs, and introduce them according to the target and injection site.

## Delivery of nanovaccines to LN

LNs distributed throughout the body are the most important lymphoid organs for vaccine-induced adaptive immunity [16, 22, 36]. Compared with the vaccine depot which slowly recruits immune cells from the periphery, LNs own high-density of APCs, B cells, and T cells. Thus, the direct delivery of vaccines to LNs harbors an enormous potential for triggering potent antibody secretion and T-cell response [37–39]. Usually, s.c. or i.m. injected nanovaccines form depots at the injection site, which migrate to LNs with the assistance of dendritic cells (DCs). Recent studies show that functionalized nanoadjuvants injected to tumors can form in situ vaccines, which are then captured by DCs and delivered to tdLNs [40, 41]. In addition, strategies to deliver drugs/antigens to LN subareas (*e.g.*, B cell follicles) have also been developed [35, 42].

### Delivery of nanovaccine from depot to LNs

#### Nanovaccines forming loose depot

**Fig. 2:**
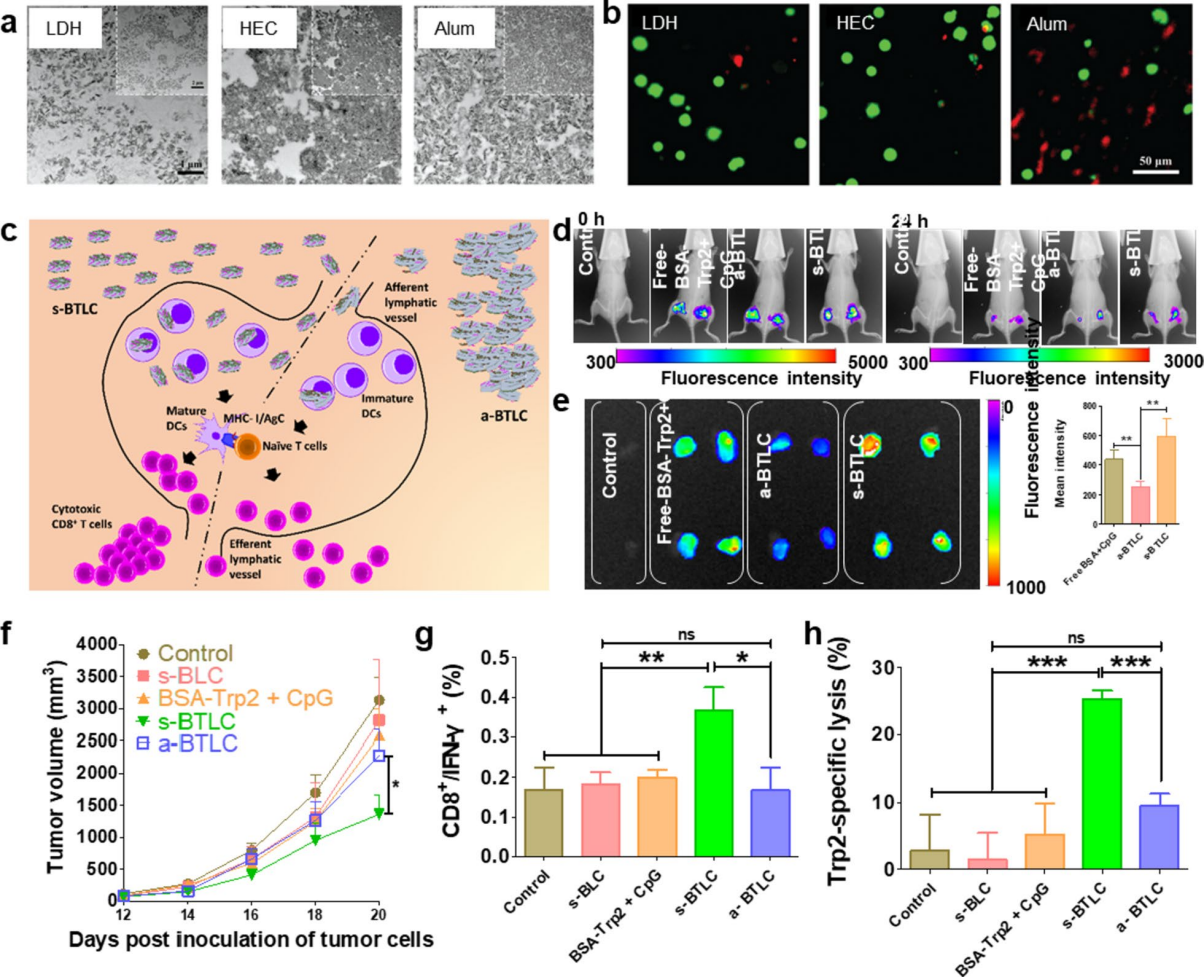
The structure of depot formed by different adjuvants. **a** Representative TEM images of the depot microstructures at day 35. **b** Representative fluorescent image of the isolated cells from different depots at day 2, with Calcein AM (green) and propidium iodide (PI, red) stained for live and dead cell, respectively. **c** Schematic illustration for migration of s-BTLC and a-BTLC from the injection site to LN. **d**, **e** Biodistribution of a-BTLC and s-BTLC in mouse (**d**) and LNs (**e**). **f** Tumor volume of mice treated with s-BTLC and a-BTLC. **g**, **h** Level of antigen-specific T cells in splenocytes derived from vaccinated mice. **a**, **b** Adapted with permission from [7]. Copyright 2018 Wiley–VCH GmbH. **c**–**h** Adapted with permission from [45]. Copyright 2018 Elsevier

Nanovaccines in the depot retain at the injection site for several months, which gradually stimulate the immune system to induce a durable immunity. However, the side effects such as local inflammation, hemolytic activity and T cell apoptosis usually happen for the clinically used adjuvants such as alum and emulsions [43, 44]. One of the most important reasons for this phenomenon is the strictly restricted migration of APCs in the dense depot. In a previous study, Chen et al*.* [7] compared the depot structure formed by layered double hydroxide (LDH), hectorite (HEC), and Alum adjuvant. As shown in Fig. 2a, the transmission electron microscope (TEM) images of the depot slices showed that the hard internal structure of Alum depot was filled with large and dense aggregates. In contrast, the LDH and HEC depots filled with smaller and lower-density microstructures formed looser structures. The further analysis showed that APCs extracted from the LDH and HEC depots are alive and mature, while that of Alum depot seemed to be damage (Fig. 2b). These results indicate that a loose depot structure is more conducive to recruit more live cells and promotes the antigen presentation. With the increase in colloidal stability, nanovaccines are easier to detach from the depot and then migrate to LNs, which has been demonstrated by a recent study conducted by Zhang et al*.* [45]. In this study, they prepared mono-dispersed (s-BTLC) and aggregated (a-BTLC) LDH nanovaccines (Fig. 2c). Both s-BTLC and a-BTLC injected subcutaneously formed a vaccine depot at the injection site, however, compared with a-BTLC, more s-BTLC nanovaccines migrated from the injection site to the LN after 24 h (Fig. 2d–e). As expected, the enhanced accumulation of s-BTLC nanovaccines in LNs promoted much stronger antigen-specific T cell responses, thereby more efficiently inhibiting the growth of melanoma than the aggregated a-BTLC nanovaccines (Fig. 2g, h). In addition, Xu et al*.* [46] showed that s.c. injection of PEGylated reduced graphene oxide nanosheet (RGO-PEG, 20–30 nm in diameter) with high colloidal stability could rapidly deliver 15–20% of the loaded neoantigens to LN and retained it for up to 72 h, achieving > 100-fold improvement in LN-targeted delivery when compared with soluble vaccines. Not only that, the direct interaction between RGO-PEG nanovaccines and DCs in LN induced intracellular reactive oxygen species (ROS), which further increased the antigen processing and presentation capacity of DCs, thereby eliciting potent and durable (up to 30 days) neoantigen-specific T cell responses to eradicate the established MC-38 colon carcinoma.

**Fig. 3:**
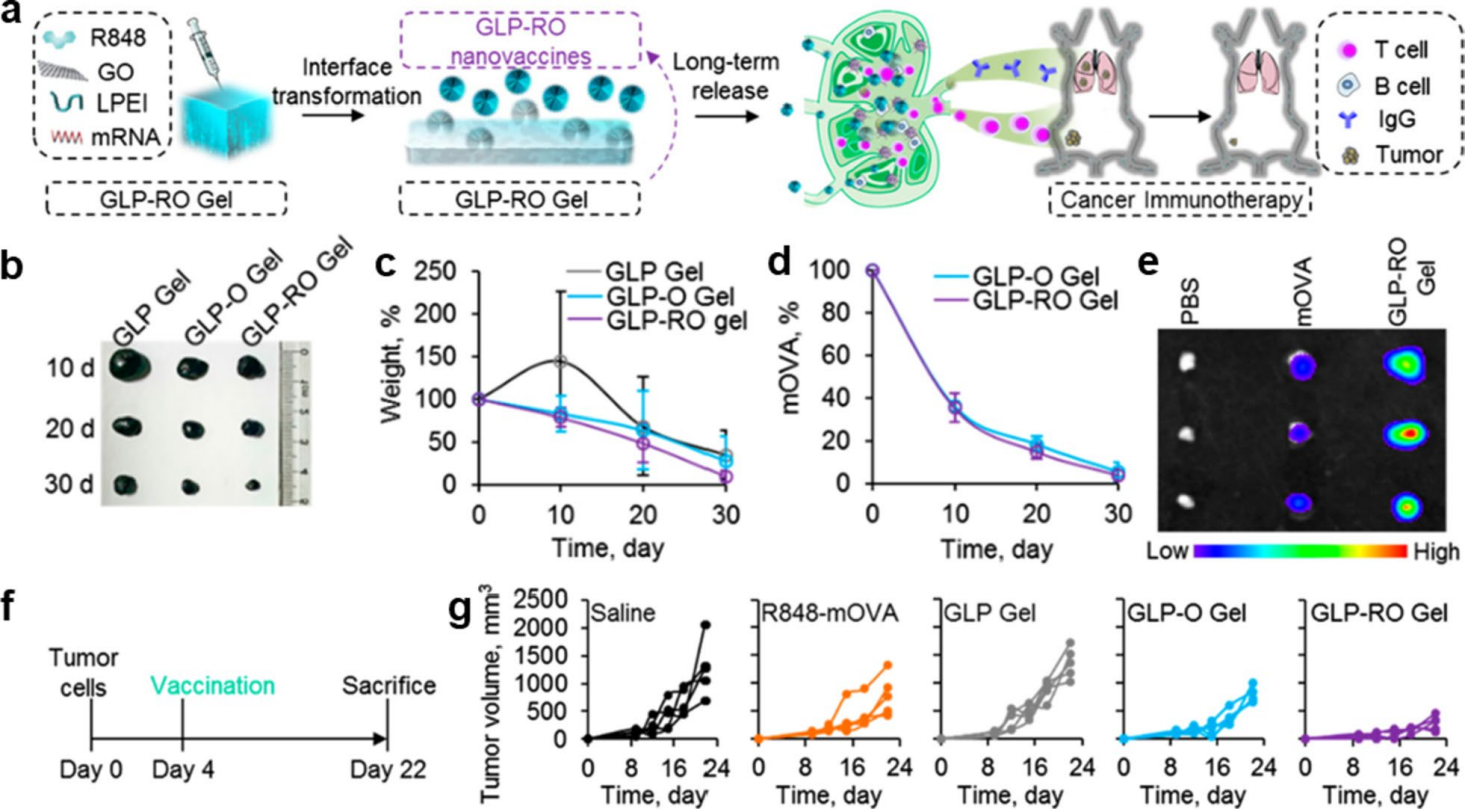
In situ transformable RNA nanovaccine for durable cancer immunotherapy. **a** Schematic illustration of the fabrication and functional mechanism of the transformable hydrogel. **b** Typical photograph and **c** weight of hydrogels collected from the mice after injected for 10, 20, and 30 days. **d** Quantitative analysis of mOVA in GLP-O and GLP-RO Gels after injected in the mice for indicated days. **e** IVIS imaging of Cy5.5 labeled mOVA in LNs after injected for 2 days. **f** Illustration of the treatment intervals. **g** The growth curves of B16-OVA tumors with various treatments. Adapted with permission from [47]. Copyright 2021 American Chemical Society

Similarly, another study conducted by Yin et al*.* [47] further demonstrated the importance of increasing the accumulation of nanovaccine in LN for enhancing cancer immunotherapy. In their study, an injectable GLP-RO Gel formed by graphene oxide (GO) and low molecular weight polyethylenimine (LPEI) was designed, and the model antigen ovalbumin mRNA (mOVA) and molecular adjuvant (R848) were encapsulated within the GLP-RO Gel through electrostatic interaction and π-π stacking (Fig. 3a). When embedded in a liquid solution, the GLP-RO gel was unstable at the interface and gradually transformed into GLP-RO NPs. Therefore, after being subcutaneously injected, the GLP-RO NPs slowly released from the GLP-RO Gel within 30 days, which then delivered R848 and mOVA to the LNs (Fig. 3b–e). The in vivo results showed that once vaccination of the transformable GLP-RO Gel significantly increased the number of antigen-specific CD8^+^ T cells and the level of OVA-specific antibody in mice, efficiently inhibiting or preventing the tumor growth or metastasis (Fig. 3f–g).

#### Nanovaccines with deformable structure

Lymphatic vessels are the main channels for transporting nanovaccines to LNs. Generally, the diameter of human initial lymph vessels is 10–60 μm, while the diameter for larger lymphatic vessels can be up to 2 mm [48, 49]. Therefore, nanovaccines with a size from nanometer to micrometer can be efficiently transported to LN through lymphatic vessels in theoretically [50]. However, the intercellular spaces of endothelial cells of human/mouse lymphatic vessels range from 20 to 100 nm, which is the gate for selecting the entrance of exogenous cargoes [51]. Therefore, the delivery of nanovaccines to LNs are highly size-dependent. The previous studies showed that nanovaccines with a size less than 100 nm (an optimal size being ~ 40 nm) could directly pass through the intercellular spaces of endothelial cells on lymphatic vessels in mice [52–54]. Nanovaccines with a size ranges from 100 to 200 nm, the filtration of nanovaccines into the lymphatic vessels may affected by their physicochemical properties (*e.g.*, size, shape, surface charge) [18, 55]. For nanovaccines that are larger than 200–500 nm, they mainly retain at the injection site [56–58], and need to be carried into the lymphatic system by APCs, which can squeeze through openings between overlapping endothelial cells [59, 60]. Feng et al*.* showed that ZnO nanovaccines ranging in size from 250 to 850 nm all induced similar levels of antibodies [61]. Chen et al*.* also compared the immune induction ability of LDH nanovaccines with different sizes [62]. In their study, they found that LDH nanovaccine with a size of 116 nm induced the strongest antibody immune response, and the larger LDH nanovaccines (243 and 635 nm) induced lower but similar levels of antibody immune response. These results indicate that the size of the nanovaccine is the key to its LN infiltration and immune-inducing ability. For vaccines that remain at the injection site, the size of the vaccine does not significantly affect the induction of immune responses.

**Fig. 4:**
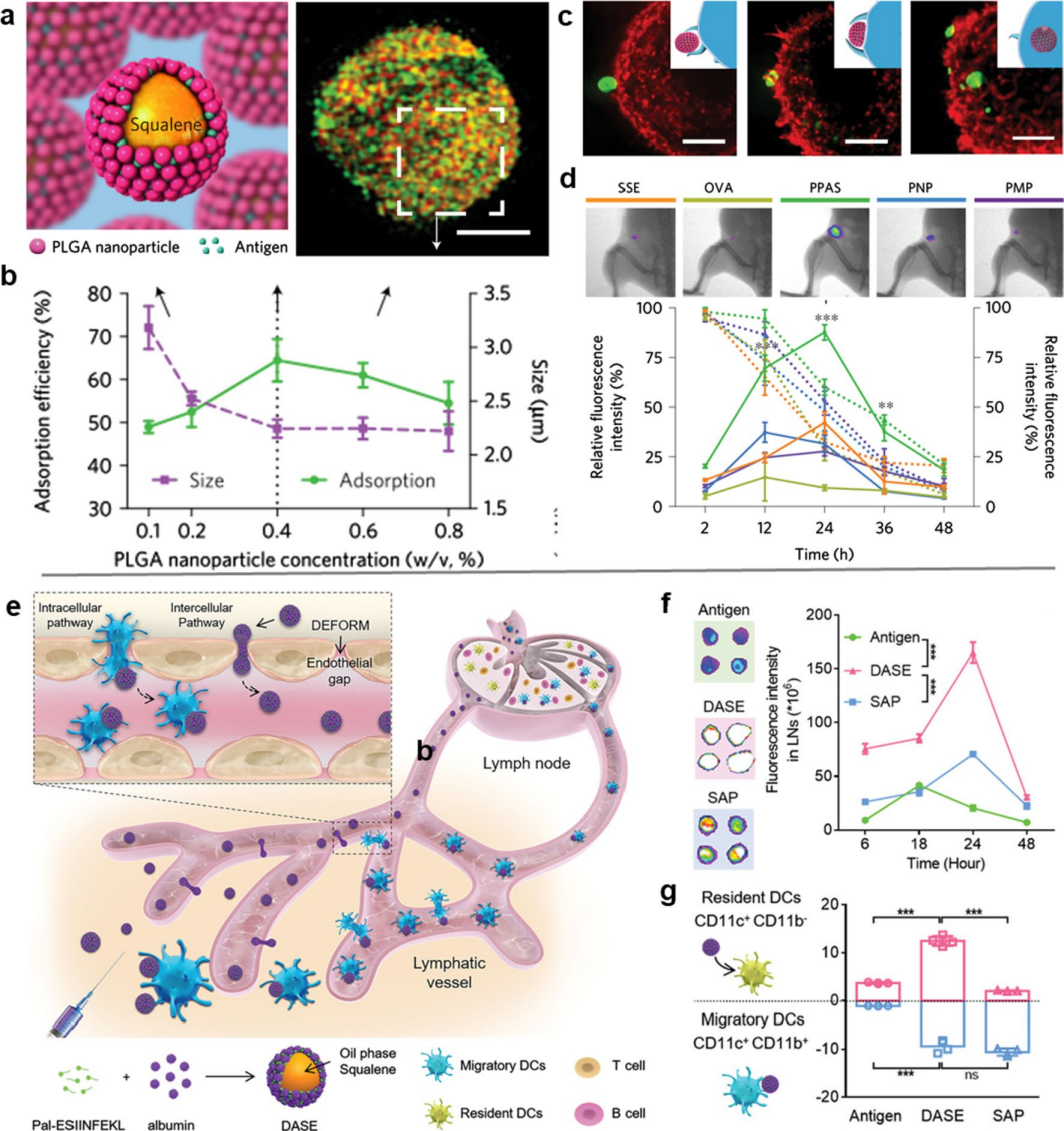
Deformable albumin-stabilized emulsions for LN-targeted vaccine delivery. **a** Schematic representation and the structured illumination microscopy (SIM) image of antigen-adsorbed PPAS droplets. The scale bar is 5 µm. **b** Particle concentration-dependent size and antigen adsorption efficiency of PPAS. **c** The process of the phagocytosis of PPAS/OVA complexes by bone-marrow DCs (membrane actin and OVA were labelled by rhodamine-phalloidin (red) and Alexa Fluor 488 (green), respectively). **d** Presence of the migrated antigens in the draining lymph nodes at 24 h, and quantitative fluorescent intensity of antigens at the injection sites (dotted line) and dLNs (solid line). **e** Schematic illustration of the deformable strategy of lymph-node transfer. **f** Antigen accumulations within the LNs 24 h after administration. The nucleus and antigens are labeled by DAPI (blue) and Cy5 (red), respectively. **g** The tendency of intercellular and intracellular pathways based on the proportion of resident DCs (CD11c^+^ CD11b^−^) and migratory DCs (CD11c^+^ CD11b^+^) within the LNs. **a**–**d** Adapted with permission from [65]. Copyright 2018 Springer Nature. **e**–**g** Adapted with permission from [23]. Copyright 2021 Wiley–VCH GmbH

Although it is believed that the accumulation efficiency of nanovaccines in LN can be improved by reducing the size, it is difficult to achieve this due to the size diversity of antigens and adjuvants. For example, the size of protein or viral subunit antigens is less than 10 nm, the size of supramolecular particulate antigens (*e.g.*, virus-like particles) ranges from 20 to 200 nm, and the size of virosomes is approximately 100–200 nm [63, 64]. After being mixed with adjuvants (such as alum, emulsions adjuvants), the vaccines tend to form larger particles or aggregates. Alternatively, increasing the softness of nanovaccines is another effective strategy to enhance the LN-targeted accumulation efficiency of nanovaccines. Xia et al*.* [65] developed a PLGA NP (PNP) stabilized Pickering emulsion adjuvant system (PPAS) that retained the force-dependent deformability and lateral mobility of presented antigens. As shown in Fig. 4a, b, the squalene emulsions were coated by PLGA NPs, and the droplet size decreased with increasing particle concentration, which corresponded with the specific surface area and exposed a greater number of surface gaps for antigen adsorption. Benefiting from the pliability, the PPAS was able to deform their structure to increase the contact area with DCs. In the binding zone, antigens were laterally streaming on the droplet surface to trigger the multivalent interactions and ultimately induce phagocytosis (Fig. 4c). The in vivo data showed that the deformable PPAS could be quickly transported from the injection site to LN by DCs, and achieved the highest accumulation of PPAS in LN than the solid NPs (Fig. 4d). As expected, the immunological data further showed that the PPAS induced the most potent anti-tumor immune responses to efficiently inhibit or prevent the growth or occurrence of lymphoma in mice. In another study, Song et al*.* showed that the soft emulsion can not only be transported to LN by DCs, but also can deform and squeeze through the endothelial space on the lymphatic vessels to reach LN (Fig. 4e) [23]. In this study, they engineered an albumin-stabilized emulsion as a deformable vaccine delivery system (DASE) by sonicating albumin, squalene, and lipopeptide OVA (palmitic acid-ESIINFEKL, Pal-ESIINFEKL). Owing to the lipid chain (palmitic acid), the Pal-ESIINFEKL was inclined to insert on the oil/water interphase during sonication, resulting in an albumin-stabilized oil-in-water emulsion with high antigen-loading efficiency. Although both solid albumin particles (SAPs) and DASE caused evident antigen depot formation, the DASE more efficiently migrated from the injection site to LNs (Fig. 4f). The underlying reason for this phenomenon is that the transport of DASE to LN was achieved through self-deformation-mediated active lymphatic infiltration and DC transport, while the delivery of SAP to LN depended only on DC. The in vivo investigation showed that the DASE treatment induced the highest level of antigen-presenting DCs in LN, among which 10.6% were resident DCs (CD11c^+^ CD11b^−^; which can only uptake antigens via direct LN-delivery by DASE), whereas 9.37% were migrated DCs (CD11c^+^ CD11b^+^; which can capture antigens at the injection site and then home to LN). In comparison, the antigen-presentation was mainly contributed by the migratory DCs in mice treated with SAP (Fig. 4g). These results indicate that with the enhancement of DASE flexibility and size optimization, the depot-mediated delivery and the direct LN transfer along the interstitial flow occurred simultaneously, greatly improving the delivery efficiency of nanovaccines.

### Delivery of in situ nanovaccine from tumor to tdLNs

Different from delivery of the established nanovaccine from the depot to LN, in situ antigens captured by nanoadjuvants can be delivered from the tumor to tdLN to induce personalized anti-tumor immune response [41, 66, 67], and more efficiently inhibit the tumor growth by reducing immune attack off-target [68–70]. Usually, the in situ nanovaccines are delivered to tdLNs with the assistance of APCs. Recent studies have shown that some nanovaccines/nanomedicines can filtrate into tumor lymphatic vessels and reach tdLNs by responsively reducing the size, or directly target tdLNs through specific surface ligands [71, 72].

#### Delivery of nanovaccine to tumor resident APCs

**Fig. 5:**
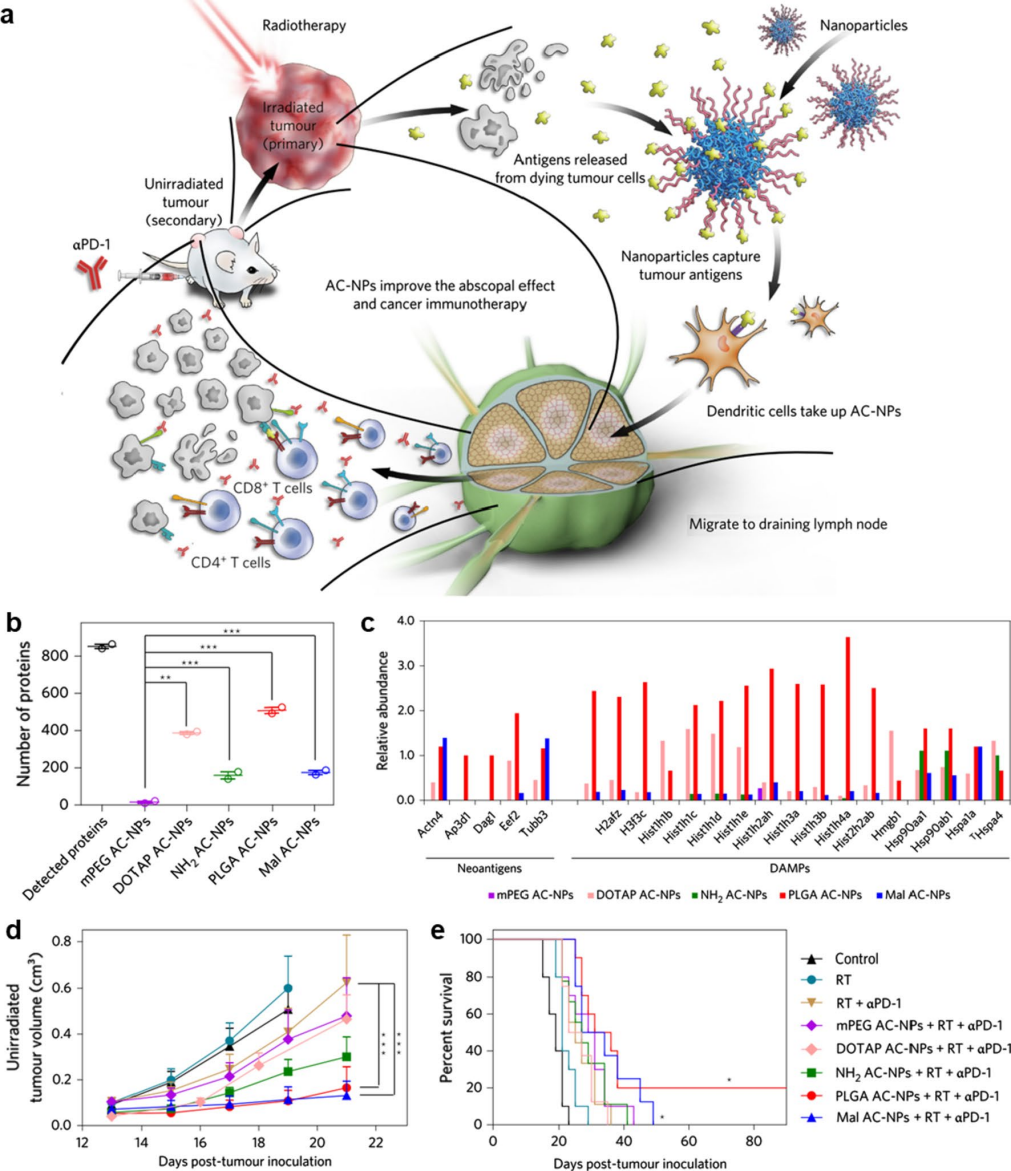
Delivery of neo-antigens to APC in tumor by functionalized nanoadjuvant. **a** Schematic depiction of utilizing AC-NPs to improve cancer immunotherapy. **b** Number of unique proteins bound to AC-NPs. **c** The relative abundance of neoantigens and damage-associated molecular pattern proteins (DAMPs) captured by AC-NPs. **d** Average tumor volume and **e** survival curves of unirradiated tumors. Adapted with permission from [40]. Copyright 2019 Nature Springer

Tumor resident APCs are the main vehicles to deliver nanovaccines from tumor to tdLNs. Min et al*.* [40] reported an antigen-capturing NPs (AC-NPs) that can deliver in situ tumor antigens to tumor resident APCs to improve cancer immunotherapy. As shown in Fig. 5, the engineered AC-NP formulations efficiently disrupted the tumors via radiotherapy, then captured and delivered tumor-specific proteins to APCs to induce potent personalized anti-tumor immunotherapy. Notably, this study also showed that the surface chemistries of AC-NP directly determines the diversity and composition of the tumor proteins captured by the AC-NPs. The PLGA and DOTAP modified AC-NP formulations captured the most-comprehensive set of proteins, while PLGA and Mal modified AC-NPs captured most neoantigens (Fig. 5b, c). The in vivo evaluation showed that PLGA and Mal modified AC-NPs significantly improve the immunotherapy and abscopal effect, and elicited the most-robust therapeutic response across all the treatment groups, denoting that reasonable design of the surface properties of functionalized nanoadjuvants is the key to achieving efficient in situ cancer immunotherapy (Fig. 5d, e). In addition, Yang et al*.* [40] designed a multifunctional nanoadjuvant assembled from doxorubicin (DOX, a chemical drug), 2-(1-hexyloxyethyl)-2devinyl pyropheophorbide-a (HPPH; a photosensitizer) (CCPS/HPPH/DOX) and chimeric cross-linked polymersomes (CCPS) for MC38 colorectal cancer immunotherapy [41]. Once the CCPS/HPPH/DOX reached tumor post i.v. injection and exposure to laser, a synergistic photodynamic therapy and chemotherapy was initiated to induce the release of TAAs, which were captured and delivered to tumor resident DCs for inducing in situ anti-tumor immunity. The in vivo data showed that DCs in tdLN were successfully activated by CCPS/HPPH/DOX, and a potent CD3^+^CD8^+^ T cell immune response was promoted to inhibit the growth of both primary and distant MC38 tumors. Similarly, Zhang et al*.* showed that LDH NPs loaded with indocyanine green (photothermal agent), DOX and CpG (Toll-like receptor 9 agonist) successfully synergized photothermal therapy, chemotherapy and immunotherapy. The in vivo data showed that the multifunctional LDH nanoadjuvant efficiently activated DCs in tdLNs and elevated the level of tumor infiltration T cells against the growth and metastasis of 4T1 breast cancer [67]. Wang et al*.* [73] presented a light-activatable immunological adjuvant (LIA), which was composed of a hypoxia-responsive amphiphilic dendrimer nanoparticle loaded with chlorin e6 to promote potent in situ anti-tumor immunity. Once the LIA was exposed to near-infrared light, molecular oxygen was rapidly consumed and generated ROS to induce the lysis of tumor cell. Meanwhile, the local hypoxic microenvironment caused the structural transformation of 2-nitroimidazole containing dendrimer to 2-aminoimidazole containing dendrimer, which activated the ‘immunological adjuvant’-like effect of the dendrimer to activate the DCs through Toll-like receptor 7-mediated signaling pathway. The in vivo data showed that the light-activatable immunological adjuvant successfully induced a robust and safe in situ anti-tumor immune response to inhibit the primary and abscopal tumor growth. It also induced a strong antigen-specific immune memory effect, preventing tumor metastasis and recurrence in 4T1 breast cancer mice. Chen et al. showed that i.t. injected polydopamine-coated and CpG-loaded Al_2_O_3_ NPs could respond to near-infrared laser irradiation to induce photothermal therapy [74]. The in vivo data shows that the functionalized Al_2_O_3_ NPs killed most of tumor tissues and triggered robust cell-mediated immune responses, thereby effectively eliminating the residual tumor cells and reducing the risk of tumor recurrence.

**Fig. 6:**
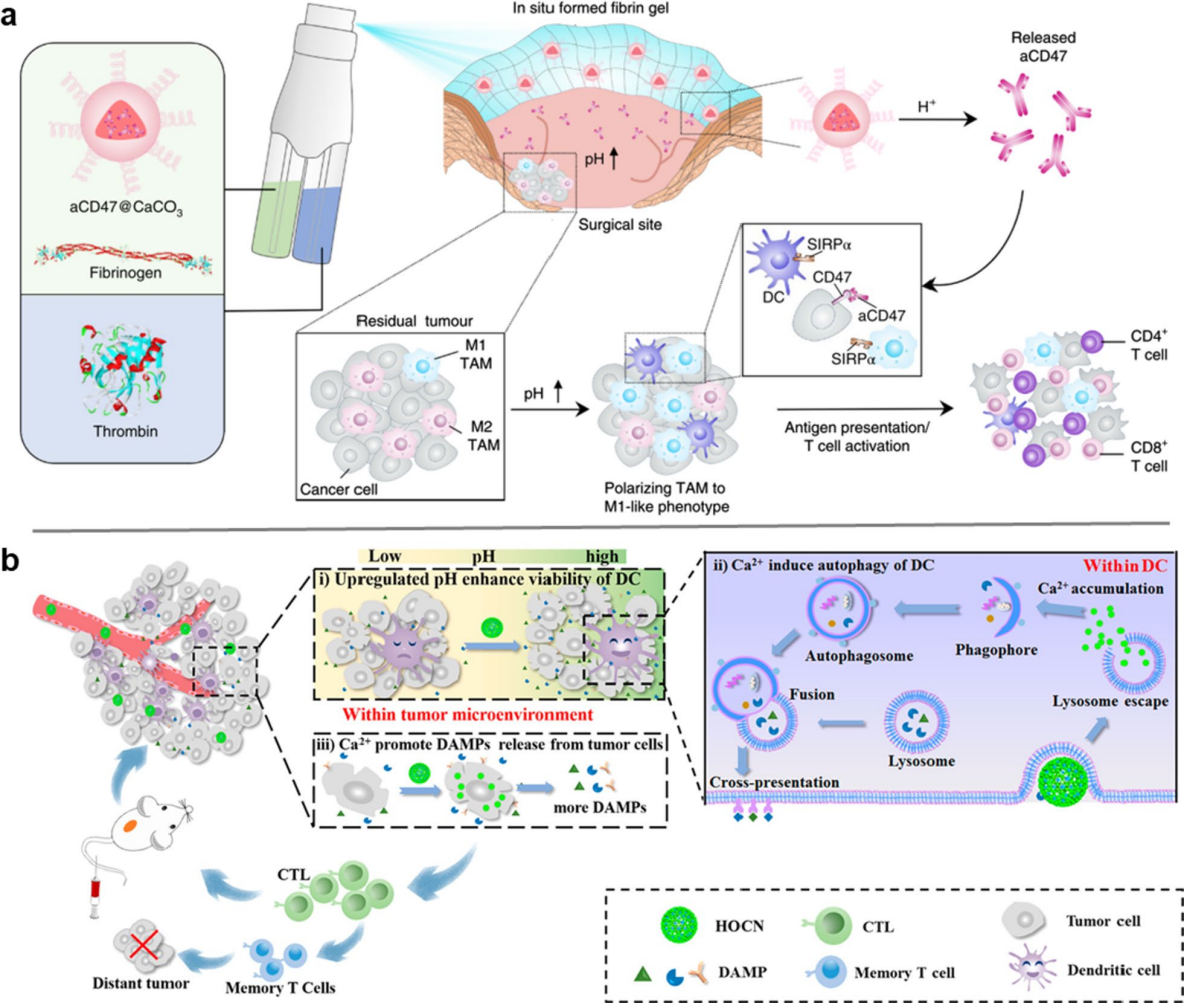
Normalization of tumor microenvironment to activate tumor resident APCs. **a** Schematic illustration of the in situ sprayed bioresponsive fibrin gel containing aCD47@CaCO_3_ nanoparticles within the post-surgery tumor bed. **b** Schematic diagram of HOCN disruption of multiple barriers in antigen cross-presentation of DCs for enhanced mitoxantrone (MTX)-mediated chemo-immunotherapy. **a** was adapted with permission from [78]. Copyright 2019 Springer Nature; **b** was adapted with permission from [79]. Copyright 2020 American Chemical Society

Neutralization of acidic tumor microenvironment is an efficient strategy to activate the tumor-associated macrophages (TAMs) and tumor resident DCs, and evoke potent personalized immune response against tumors [75–77]. Chen et al*.* [78] designed a gel encapsulated with CaCO_3_ NPs to modulate the functions of TAM and enhance cancer immunotherapy (Fig. 6a). CaCO_3_ NPs loaded with the anti-CD47 antibodies (aCD47@CaCO_3_) were dispersed in the fibrinogen solution, and formed fibrin gel at the tumor surgical site through the interaction between fibrinogen and thrombin. The aCD47@CaCO3 NPs encapsulated in fibrin gel effectively scavenged H^+^ in the surgical wound site to promote the polarization of TAMs from pro-tumoral M2-like phenotype to anti-tumoral M1-like phenotype, and the released aCD47 successfully shield CD47 protein (‘don’t eat me’ signal) expressed on the surface of tumor cells. Then the activated M1-like TAMs efficiently killed tumor cells and presented the in situ TAAs to immune system, successfully gel ‘awakening’ the host innate and adaptive immune systems to inhibit and prevent tumor recurrence and metastatic spread. Similarly, An et al*.* designed an honeycomb CaCO_3_ NP (denoted as HOCN, OVA used as skeleton) as calcium ion nanogenerator to strengthen the antigen-presentation capacity of tumor resident DCs (Fig. 6b) [79]. After mitoxantrone-mediated chemotherapy, i.v. injection of HOCN elevated the pH of the tumor microenvironment via acid hydrolysis, which efficiently improved cell viability of DCs. Ca^2+^ released from the HOCN induced autophagy in DCs to facilitate the antigen cross-presentation. Meanwhile, the intracellular release of Ca^2+^ further promoted the release of DAMPs from tumor cells into the tumor microenvironment to adjuvant DC activation, thereby greatly improving the therapeutic of cancer chemo-immunotherapy.

#### Delivery of nanovaccine to tdLNs

**Fig. 7:**
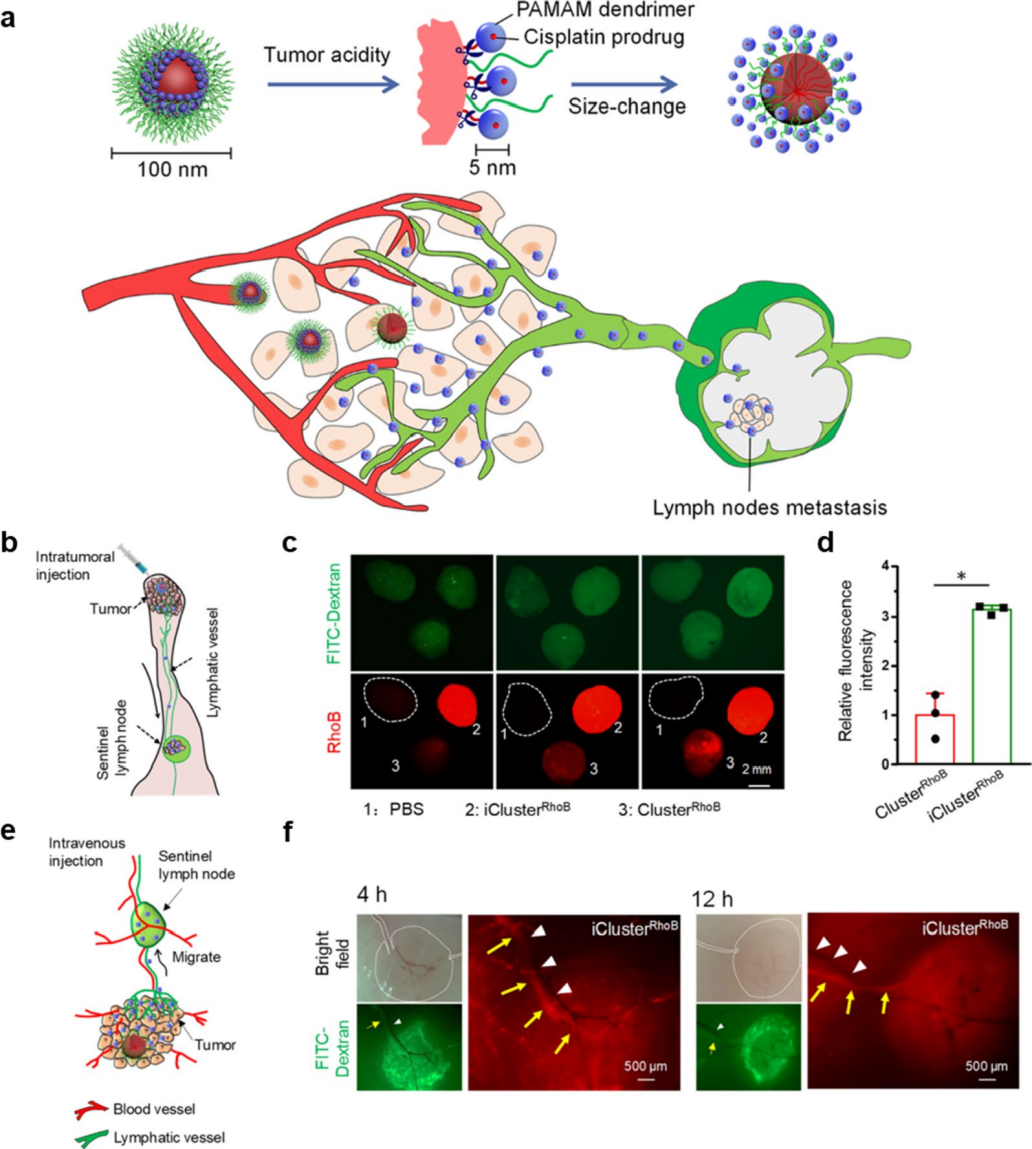
Delivery of nanoparticles from tumor to tdLNs by dynamic nanostructures. **a** Schematic illustration of tumor acidity triggered size change of iCluster and its translocation from primary tumor to LNs via tumor lymphatics. **b** Schematic illustration of the i.t. injection of iClusters. **c** Stereoscopic fluorescence microscope image of PAMAM accumulation in SLN 12 h after injection. **d** Quantitative analysis of fluorescence intensity of RhoB in SLN. **e** Schematic illustration of the i.v. injection of iClusters. **f** In vivo images of NP draining into SLNs via primary tumor lymphatic vessels (yellow arrow) after i.v. injection of iCluster after 4 and 12 h. Blood vessel (white arrow). Adapted with permission from [71]. Copyright 2019 American Chemical Society

By modifying the LN targeting ligands on the surface of the functionalized nanoadjuvant, the migration efficiency of the in situ nanovaccine from tumor to tdLNs is greatly improved. Jiang et al*.* [80] showed that a monoclonal anti-body (MHA112) that can specifically bind with peripheral lymph node address protein (PNAd) highly expressed in LN or tumor stroma was found can greatly enhance the targeting efficiency of antibody-conjugated drugs to tumor and tdLNs simultaneously. Similarly, Wang et al*.* [72] introduced nuclei isolated from tumors into activated macrophages to produce chimeric exosomes, which specifically homed to LNs and tumor to locally prime T cell activation and induce regression in primary solid tumor mouse models. Interestingly, nanovaccines with dynamic structure may actively migrate from tumor to tdLNs by responding to the acidic tumor microenvironment. For instance, Liu et al*.* [71] designed an iCluster nanoplatform, which underwent size reduction from ∼ 100 to ∼ 5 nm in the acidic tumor microenvironment, markedly promoted the diffusion of NPs into the tumor lymphatics and migration into LNs (Fig. 7a). The iCluster was prepared by attaching polyamidoamine (PAMAM, ∼ 5 nm) dendrimer onto a large NP (∼ 100 nm) via a tumor-acidity-responsive amide bond, and a non-responsive Cluster was used as a control. The iCluster was stable in blood circulation but enabled rapid release of PAMAM upon acidic environment. As shown in Fig. 7b–d, when the iCluster or Cluster were injected i.t., ∼3.5-fold higher level of the Rhodamine B (RhoB) fluorescence in sentinel LN (SLN) was observed. When the iCluster or Cluster were injected i.v. into the mice tail vein, the red fluorescence of PAMAM released from iCluster accumulated in the SLNs at 4 and 12 h post-NP injection, while no red fluorescence was observed in SLNs of Cluster treated mice (Fig. 7e–f). These studies denote that by rationally designing the physicochemical properties of the functionalized nanoadjuvant, it is possible to achieve efficient delivery of antigens or adjuvants from tumors to tdLNs.

### Delivery of nanovaccine to subareas of LN

**Fig. 8:**
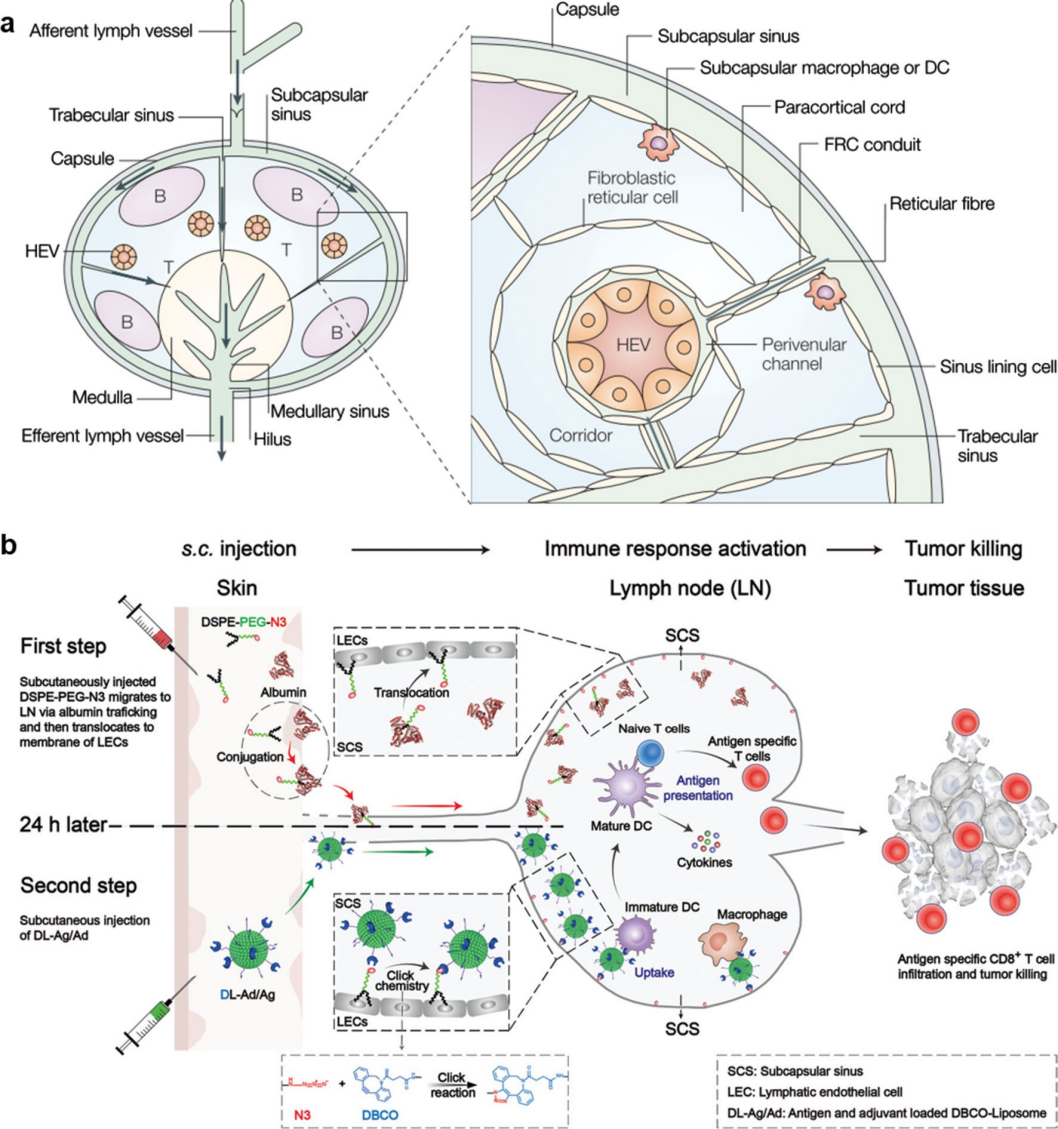
Delivery of vaccines to SCS of LN. **a** Structure of LN. **b** Schematic illustration of the click-chemistry-mediated active LN accumulation cancer vaccine system for improved vaccine delivery. **a** Adapted with permission from [11]. Copyright 2003 Springer Nature. **b** Adapted with permission from [91]. Copyright 2021 Wiley–VCH GmbH

After reaching LN, the distribution of nanovaccines in the subareas of LN also influences the strength and quality of induced immune response. Histologically, the LN can be divided into two main regions, *i.e.*, the cortex and the medulla. The cortex is composed of paracortex (T-cell area) and B-cell area that consists of B-cell follicles and (after antigen challenge) germinal centres (Fig. 8a) [11]. B-cell follicles are the main site for inducing humoral responses, whereas the paracortex is the site where circulating lymphocytes enter the LNs and where T cells interact with DCs [81–83]. The medulla contains B cells, antibody producing plasma cells (migrate from the cortex) and macrophages [84]. Nanovaccines are transported into LN through afferent lymph vessels to lymph-filled subcapsular sinus (SCS), which is adjacent to T-cell area and demarcated by lymphatic endothelial cells (LECs). Nanovaccines with a large size (> 30 nm) are scavenged by subcapsular macrophage or captured and presented to T cells by DC, while the smaller nanovaccines may directly diffuse through the gaps between LECs to reach the cortex or paracortex, then interact with B or T cells [83, 85–90].

Recently, Qin et al*.* [91] developed a click-chemistry-based active LN accumulation system (ALAS) to improve the delivery efficiency of antigen and adjuvant to the SCS of LNs. As shown in Fig. 8b, the delivery of the ALAS was divided into two steps. In the first step, the surface of LECs was modified with an azide group by injecting DSPE-PEG-N3. The DSPE-PEG-N3 hitchhiked on interstitial albumin leaking out from blood vessels and recycling back to veins via the lymphatic system through the high affinity of DSPE group to albumin. Then, the DSPE-PEG-N3/albumin complex largely migrated to the SCS of LNs, followed by a translocation from albumin to the cell membrane of LECs. The decoration of the DSPE-PEG-N3 on LECs provided targets for dibenzocyclooctyne (DBCO)-modified, antigens and adjuvant-loaded liposomes (DL-Ag/Ad). In the second step, the DL-Ag/Ad were subcutaneously injected at the same site as the DSPE-PEG-N3 injection. Since the DBCO could react with N3 modified on the LEC, DL-Ag/Ad nanovaccines migrated from the injection site to SCS of LNs were anchored on LECs, thus the accumulation of DL-Ag/Ad in LNs was greatly enhanced to improve the antigen-uptake by SCS DCs. Besides, Wang et al*.* showed that the rational surface modification also greatly enhanced the LN retention ability of nanovaccines [92]. In this study, TLR-7 agonist imiquimod (R837) was loaded into mesoporous polydopamine (MPDA) nanocarriers, and the surface of the nanovaccines was modified by polyvinyl pyrrolidone (PVP) to enhance the lymphatic drainage ability. The in vivo study showed that the PVP-modified nanovaccines efficiently accumulated in the SCS and interfollicular areas proximal LNs post 24 h injection, suggesting that the appropriate surface modification greatly enhanced the transportation and retention ability of nanovaccines in LN.

**Fig. 9:**
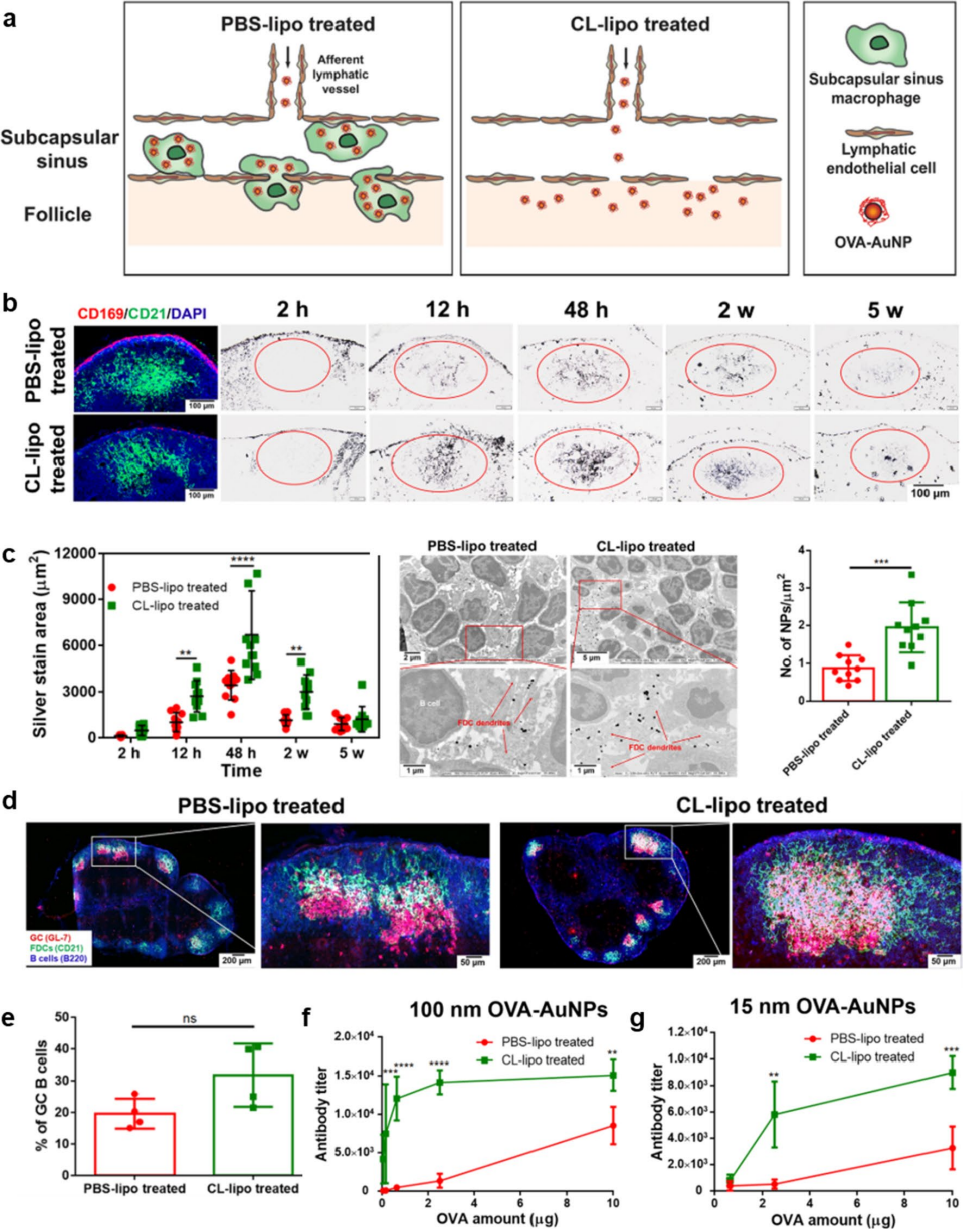
Delivery of vaccines to B-cell follicles in LNs. **a** Schematic of nanovaccine transport with and without SCS macrophages (PBS-lipo: PBS liposome, CL-lipo: clodronate liposome). **b** Histological images of B-cell follicles 7 days after intradermal footpad injection of PBS or clodronate liposome treatment. **c** Quantification and imaging of 100 nm OVA-AuNP nanovaccine accumulation in follicles. **d**, **e** Assessment of (**d**) germinal center formation and (**f**) percentage of germinal center B cells. **f**–**g** Measurements of OVA-specific antibody production after administration of (**f**) 100 nm and (**g**) 15 nm OVA-AuNP nanovaccine with (PBS-lipo treated) and without (CL-lipo treated) SCS macrophages. Adapted with permission from [42]. Copyright 2020 American Chemical Society

Aiming to induce more potent cellular or humoral responses, nanovaccines in SCS must escape from the elimination of macrophages and diffuse through the LECs to deliver more antigens or adjuvants to T or B cells. Zhang et al*.* showed that deletion of SCS macrophages promoted more nanovaccines diffused from SCS to B-cell follicles [42]. They revealed that SCS macrophages existed in the SCS macrophages played a barrier role to prevent OVA-gold NPs (OVA-AuNP) nanovaccines from accessing B-cell follicles by sequestering NPs in all locations (Fig. 9a). To overcome this problem, clodronate liposome that can specifically delete SCS macrophages was utilized to enhance the B-cell follicle-targeted delivery of OVA-AuNP nanovaccines. The in vivo data showed that the clodronate liposome successfully deleted most SCS macrophages (red) in the SCS area whereas the follicular DCs (green) in B cell follicles were remained intact (Fig. 9b). As expected, the pre-treatment of clodronate liposome enabled more OVA-AuNP nanovaccines to cross the gaps between the LECs and access B-cell follicles, generating two times more B cells (GL7^+^B220^+^) in germinal centers (red color) than that of treated by PBS liposomes (Fig. 9d–e). On this basis, the further investigation showed that 100 nm OVA-AuNP nanovaccines that were easily internalized by SCS macrophages efficiently induced 2–60 times higher level of OVA-specific antibody in mice pre-treated with clodronate liposomes than that without treatment (Fig. 9f–g), suggesting that direct delivery of nanovaccines to B cell area is a promising strategy to enhance the strength of humoral immunity.

**Fig. 10:**
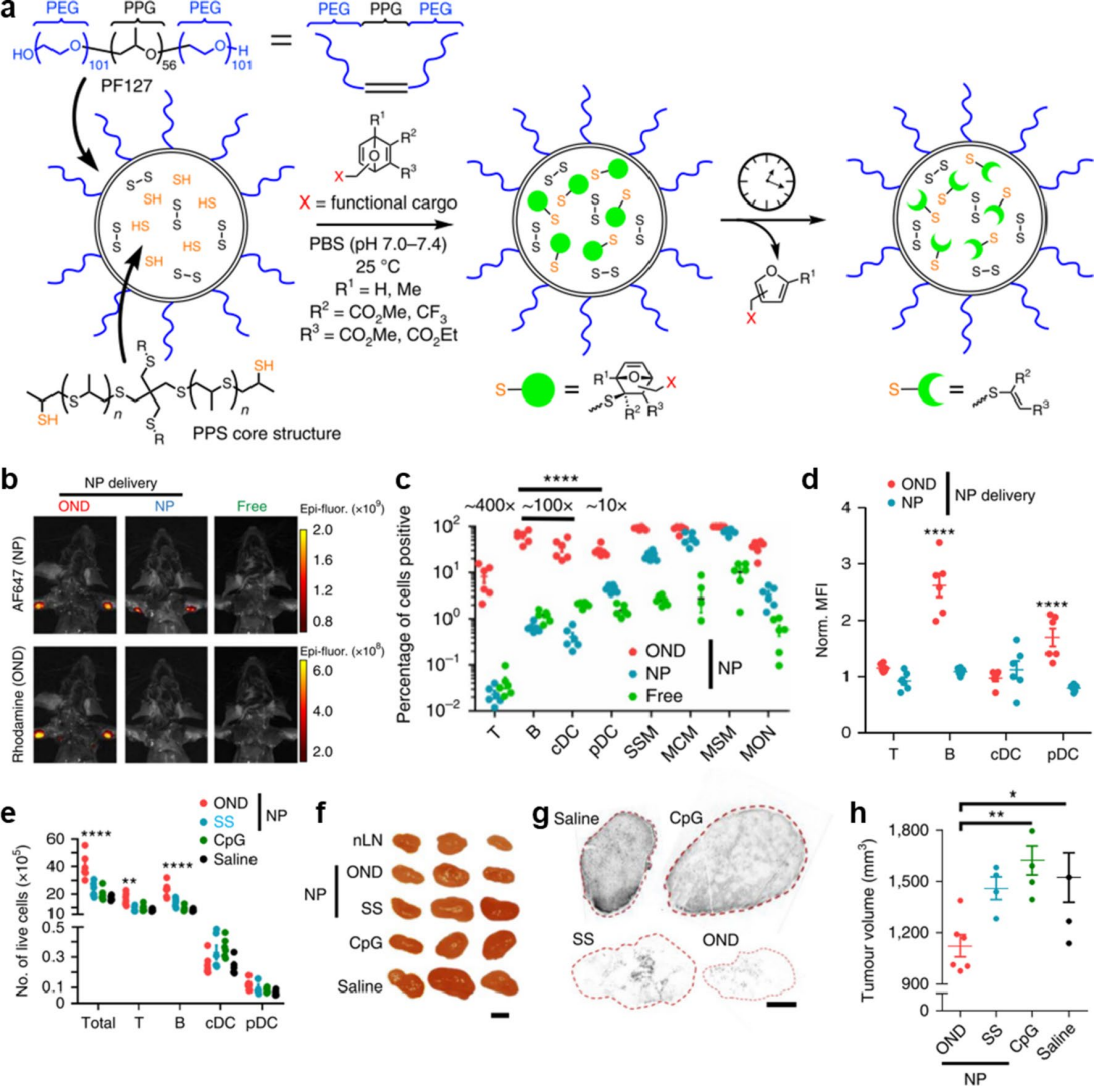
Programmable multistage nanovaccine for controllable delivery in subares of LN. **a** Schematic representation of PPS NP preparation, conjugation with OND electrophiles and retro-Diels–Alder release of furan-tagged cargo. **b** Representative fluorescence images of mice after i.d. injection of OND, NP and free rhodamine. The PPS NP was labelled with Alexa647-maleimide (AF647, non-cleavable linker) and the OND was labelled with 3-Rhod (Rhodamine, OND). **c** Percentages of LN cells of various types positive for OND (red) versus free rhodamine (Free, green) and AF647 (NP, blue) measured by flow cytometry. **d** Normalized mean fluorescence intensity (MFI) of rhodamine (red) and AF647 (blue) in the indicated LN immune cells after NP, OND or free rhodamine treatment. **e** Total numbers of LN cells of various types 24 h after i.d. treatment with NP-OND-CpG (OND), CpG disulfide bonded to NP (SS), free CpG oligonucleotide (CpG) and saline as negative control. **f**–**h** Results at day 12 after tumour implantation, following treatment for five days (starting at day 4) with formulations in (**e**). **f** Representative LN images. **g** Representative TCRVβ12 staining of sectioned Ln tumours. **h** Primary tumour size day 12 post treatment. Adapted with permission from [35]. Copyright 2020 Springer Nature

In addition, by rationally optimizing the surface chemistry, more nanovaccines may accumulate to the B/T cell area. Schudel et al*.* designed a synthetic NPs carrier system for enhanced lymphatic uptake and transport to the LN that can release its payload at different rates, thereby altering access to LN structures and tuning the amount of small- and medium-sized molecules delivered at the site of interest [35]. As shown in Fig. 10a, the thiolated poly(propylene sulfide) (PPS) NPs that can be easily taken up by lymphatic vessels and accumulate in LNs after peripheral administration were conjugated with thiol-reactive oxanorbornadiene (OND) linkers (the half-lives of which range from hours to days according to a first-order retro-Diels–Alder mechanism in a pH- and solvent-insensitive manner). The in vivo data showed that both PPS NP and PPS-OND NP (abbreviated as OND) efficiently migrated to LN after injection (Fig. 10b). However, compared to the PPS NP, more OND were taken up by various immune cells in LN, among which most of the OND were internalized by B cells and plasmacytoid DCs (pDCs; Fig. 10c, d). When TLR-9 agonist CpG was delivered by OND, the number of T, B, conventional DCs (cDCs) and pDCs in the LN increased at least threefold higher than that delivered by PPS NP (Fig. 10e), thus OND resulted in a nearly complete loss of LN EL4 tumor burden and a reduction of the size of the primary EL4 tumor (Fig. 10f–h). These results indicated that the precise delivery of adjuvant or antigens to the specific subtypes of LN cells is an efficient strategy to improve cancer immunotherapy.

## Delivery of nanovaccines to spleen

It has been shown that effective delivery of nanovaccines to LN can greatly enhance cancer immunotherapy [50, 71], but the limited number of immune cells in LN has become a bottleneck that hinders the speed and strength of the induced anti-tumor immune response [31, 93]. Alternatively, spleen, the largest secondary lymphoid organ which owns the highest density of APC and B/T cells, has been utilized as the target for vaccine delivery to enhance cancer immunotherapy [94, 95]. It has been shown that direct delivery of large amounts of vaccines to spleen successfully activate high density of immune cells and thus inducing stronger immune response [96].

### Size-dependent delivery of nanovaccines

**Fig. 11:**
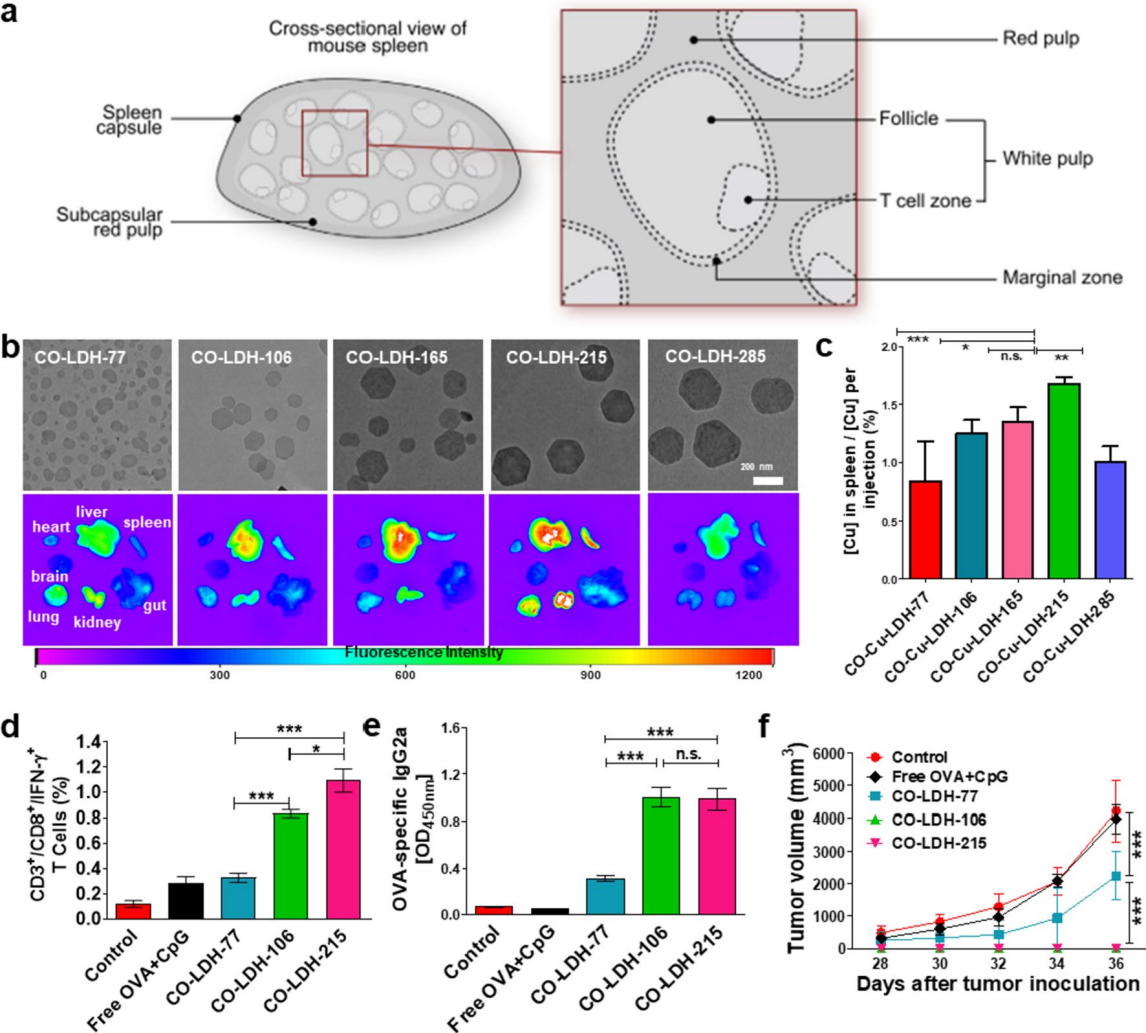
Size-dependent capture of nanovaccine by spleen. **a** Schematic illustration of the spleen structure. **b** Morphology and biodistribution of CO-LDH nanovaccines with different size in spleen. **c** Spleen enrichment efficiency of CO-LDH in spleen detected by ICP-MS. **d** The level of antigen-specific CD3^+^CD8^+^IFN-γ^+^ T cells in splenocytes. **e** The level of antigen-specific IgG2a antibodies. **f** The volume of tumor in mice treated with CO-LDH nanovaccines with different size. **a** Adapted with permission from [94]. Copyright 2013 Elsevier. b–f Adapted with permission from [25]. Copyright 2021 Springer Nature

As shown in Fig. 11a, spleen is composed of red pulp (for filtering blood and recycling iron from aging RBC) and white pulp (contains area rich in B and T cells), which are separated by an interface called the marginal zone (MZ, contains APCs). Blood flows through afferent arterial into red pulp and ends in sinusoid spaces (around the white pulp), then returns to efferent splenic veins [94]. Before reaching the B or T cell zone, nanovaccines need to pass through three barriers after i.v. administration, i.e., liver Kupffer cells (KCs) barrier, splenic red pulp macrophage barrier and MZ barrier. Generally, nanovaccines with a size of less than 100 nm are mainly captured and removed by liver KCs, while nanovaccines with larger sizes are filtrated by red pulp and scavenged by the splenic red pulp macrophages [97–99]. Only those between 100 and 200 nm in size and successfully escaped from macrophages elimination can pass through the MZ and finally enter the B or T cell area [98, 100, 101]. On this basis, Zhang et al*.* [25] investigated how the size of nanovaccine affects their splenic accumulation and anti-tumor immune induction efficiency. To achieve this aim, they engineered a plate-like nanovaccine by loading model antigen OVA and Toll-like receptor 9 agonist CpG onto the surface of LDH NPs, which has a size ranges from 77 to 285 nm, to prepare CO-LDH-n (n denotes the size of LDH). The in vivo study showed that CO-LDH-215 nanovaccines most efficiently accumulated in the spleen than other nanovaccines (Fig. 11b, c). Moreover, the immunological analysis showed that CO-LDH-106 and CO-LDH-215 nanovaccines induced higher level of cytotoxic T cells and IgG2a antibody to delay the growth of lymphoma in mice than the smaller nanovaccines (Fig. 11d–f). It is worth noting that CO-LDH-215 nanovaccines induce the strongest immune response compared to the smaller LDH nanovaccines, even though they can more easily pass through the MZ area to reach the B or T cell area. This means that most of the nanovaccines in this study fail to escape the elimination of red pulp macrophages, and the dose of the nanovaccine accumulated into the spleen may determine the strength of the induced anti-tumor immune response.

**Fig. 12:**
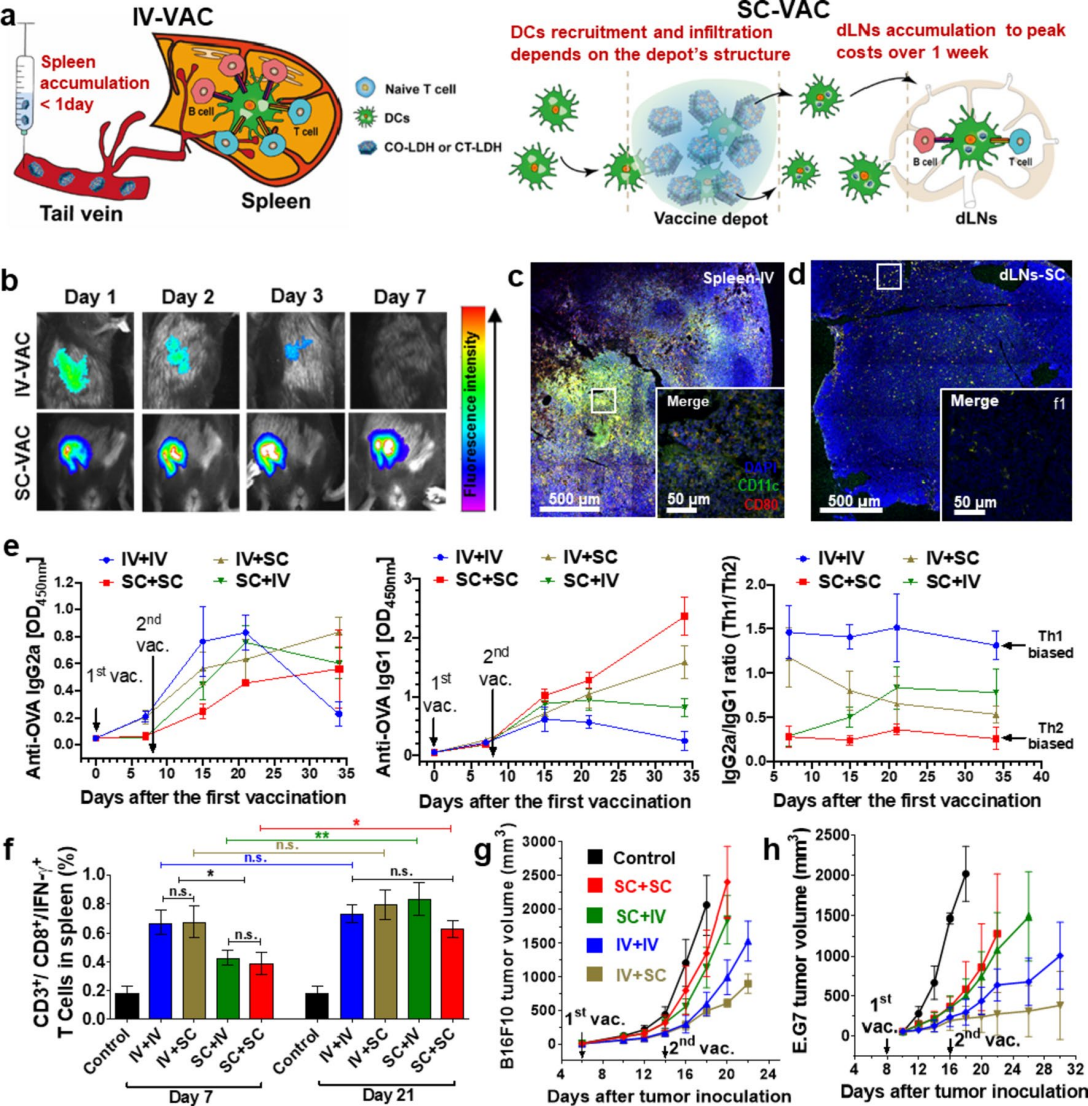
Spleen-targeted delivery of nanovaccines. **a** Schematic illustration for vaccine delivery processes after IV and SC vaccination, respectively. **b** Biodistribution of CO-LDH nanovaccines. **c**, **d** The localization of CD11c^+^ (green) DCs and CO-LDH (grey), and maturation of DC (marker CD80^+^, red) in spleen and LN collected from mouse with IV and SC vaccination, respectively. **e** The time-dependent levels of specific anti-OVA antibodies (IgG1, IgG2a). **f** The level of antigen-specific CD3^+^CD8^+^IFN-γ^+^ T cells in splenocytes. **g**, **h** The tumor volume of B16F10 melanoma and E.G7-OVA lymphoma mice. Adapted with permission from [9]. Copyright 2020 Elsevier

In another study, Zhang et al*.* employed a classical “priming + boosting” vaccination strategy to investigate whether the change of vaccination route will affect the induction of anti-tumor immune responses (Fig. 12a) [9]. In this study, four vaccine routes include twice i.v. injection (IV + IV; vaccine targets to spleen), twice s.c. injection (SC + SC; vaccine targets to LNs), and combined i.v. and s.c. injection (IV + SC or SC + IV; vaccine targets to spleen and LN) were used. As shown in Fig. 12b–d-IV injected CO-LDH nanovaccines rapidly enriched into spleen within 1 day and retained in spleen for at least 3 days, while the SC-injected CO-LDH nanovaccines mainly retained at the injection site and few of them were observed in draining LNs (dLNs). Moreover, IV + IV vaccination rapidly induced potent T helper (Th) 1-polarized anti-tumor immune responses within 7 days, but the strength of the immunity dropped 2 weeks after the last vaccination. In comparison, the same nanovaccines received SC + SC vaccination took 2–3 weeks to gradually induce durable anti-tumor immune response. As expected, the in vivo data showed that the nanovaccines administrated by IV + IV route more efficiently inhibited the growth of melanoma and lymphoma tumors than by SC + SC route (Fig. 12g, h). Interestingly, the “IV-priming + SC-boosting” vaccination combination was also noticed could rapidly induce potent and durable anti-tumor immune responses, which most efficiently inhibited the growth of early-stage melanoma and lymphoma tumors, with the tumor volume reduced by > 75–90% in comparison with the control group (Fig. 12e–h). These results indicate that delivering the same vaccine to the spleen will greatly enhance cancer immunotherapy, and the rational optimization of the vaccination schedule will maximize the therapeutic effect of the vaccine.

### RBC-based delivery of nanovaccines

**Fig. 13:**
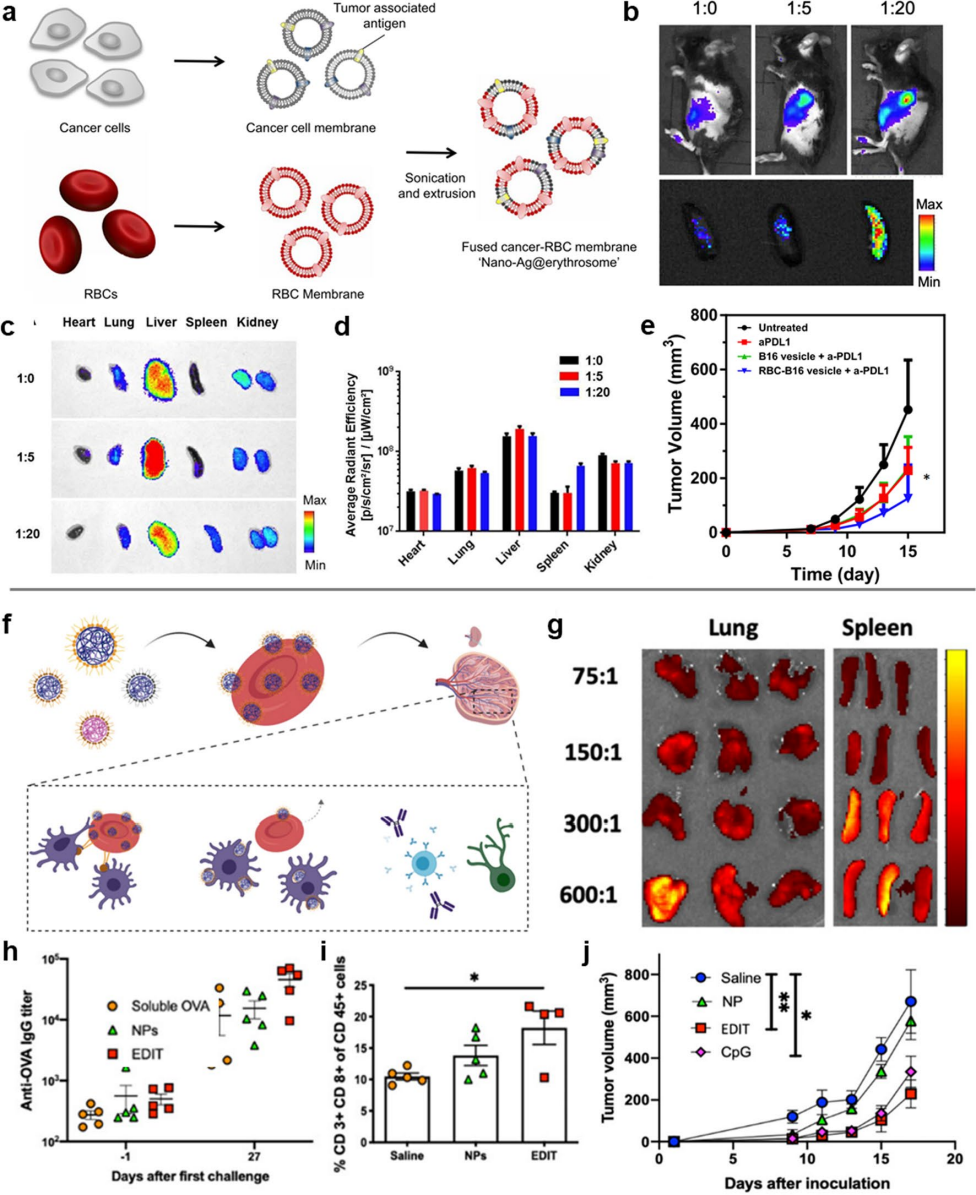
Spleen-targeted erythrocytes-based nanovaccines. **a** Preparation of nano-Ag@erythrosomes by fusing tumor antigen–associated cell membrane into nanoerythrosomes. **b**, **c** Biodistribution and ex vivo imaging of organs 1 h after intravenous injection of nano-Ag@erythrosomes at various ratios and **d** corresponding quantification results. **e** B16F10-luc tumor growth curve after mice were treated with nano-Ag@erythrosomes or B16-membrane vesicle plus aPDL1. **f** Schematic for engineering a handoff of nanoparticles at the spleen via erythrocyte hitchhiking. **g** In vivo fluorescence images of lungs and spleen harvested from mice 20 min after being injected with erythrocytes incubated at different nanoparticle-to-erythrocyte ratios. **h, i** Analysis of the (**e**) anti-OVA IgG titer and **f** the CD3^+^ CD8^+^ cells in the spleen. **j** Tumor growth curves for mice inoculated after prophylactic vaccinations by different treatment groups. **a**–**e** Adapted with permission from [31]. Copyright 2019 The Author(s). **f**–**j** Adapted with permission from [104]. Copyright 2020 PNAS

Although the successful delivery of nanovaccines to spleen has been proved can efficiently promote potent anti-tumor immunity, most of the nanovaccines (~ 98–99%) were captured and eliminated by liver KCs and splenic red pulp macrophages [42]. On this consideration, high dosage of nanovaccine is required to ensure enough nanovaccines are delivered to spleen [102]. However, it is worth noting i.v. administration of nanomaterials at a high dosage and frequency may accelerate tumor metastasis, even those have been demonstrated with high biocompatibility [103]. Therefore, novel vaccine delivery system such as RBC-based nanovaccines that are derived from the host and can escape from macrophage elimination have been developed for cancer immunotherapy. Han et al*.* reported an antigen delivery system based on the nanoerythrosomes derived from RBCs [31]. As shown in Fig. 13a, the nanoerythrosomes were prepared by fusing ghost RBCs membrane and tumor cell membrane with varied RBC membrane-to-tumor cell membrane (R:T) ratio. At a high R:T ratio, more tumor antigen accumulated in the spleen but not in the liver and other organs, while at lower R:T ratios, nanoerythrosomes mainly accumulated in the liver (Fig. 13b–d). When the nanoerythrosomes were combined with PD1 antibody (aPD1) for the treatment of B16F10-luc tumor, it was found that nanoerythrosomes significantly increased the tumor suppression efficacy (Fig. 13e). These results indicate that RBC-derived nanoerythrosome is an ideal nanoplatform for enhanced delivery of tumor antigens to spleen, which effectively triggers a strong anti-tumor immune response. In another study, Ukidve et al*.* [104] engineered a hitchhiking system erythrocyte-driven immune targeting (EDIT), which induced the delivery of the attached NPs predominantly to the splenic APCs instead of lungs to achieve cellular and humoral immunity (Fig. 13f). As shown in Fig. 13 g, the model antigen OVA was capped on the surface of 200 nm polystyrene carboxylate (PS-COOH) to generate protein-capped NPs, which were then attached to erythrocytes to obtain EDIT. When the NP:erythrocyte was at a high ratio of 300:1, more NPs were delivered to spleen by EDIT (Fig. 13g). As expected, EDIT delivered more NPs to spleen, efficiently activated the immune system and induced stronger anti-tumor cellular and humoral immune response, successfully delaying the growth of E.G7-OVA lymphoma (Fig. 13h–j).

## Conclusion

In summary, nanovaccines are indisputably at the forefront against malignant tumors, and the enhancement of lymphoid organ-targeted delivery of nanovaccines provide an appealing concept for the efficient cancer immunotherapy. Recent advances have witnessed tremendous contribution of the efficient delivery of nanovaccines to lymphoid organs in strengthening cancer immunotherapy. For instance, optimized physical characteristics (*e.g.*, size, colloidal stability, electrostatic interaction, deformability) or chemical properties (*e.g.*, light-, pH- and enzyme-responsiveness) of nanovaccines, enabling them to deliver more antigens from the injection site or tumor to LNs, or from blood to spleen. Moreover, through rationally tailoring surface ligands, nanovaccines can more effectively migrate to specific subareas of LN to activate immune cells, thereby resulting in stronger anti-tumor immune response. Furthermore, extend the circulation of nanovaccines and prevent them from macrophage elimination also greatly enhance the accumulation of nanovaccines in lymphoid organs. Meanwhile, simultaneous delivery of nanovaccines to LNs and spleen rapidly induce potent and durable anti-tumor immune response. Collectively, the improvement of the lymphoid organ-targeted delivery efficiency of nanovaccines has greatly improved the therapeutic efficacy of nanovaccines without increasing the formulation complexity.

Although comparable high delivery efficiency of nanovaccine to lymphoid organs have been achieved by different strategies, it is worth noting that the therapeutic efficacy is still challenged by the suboptimal PK and potential toxicity of the nanovaccines. In general, nanovaccines are readily combined with albumin and opsonin in tissue fluid or blood, which makes them easy to be swallowed and eliminated by macrophages [33, 105]. Especially for the nanovaccines delivered to spleen, liver KCs and splenic red pulp macrophages are the major obstacles in preventing them from reaching MZ or B/T cell area [33, 34]. To overcome this shortage, the dosage and injection frequency are required to be increase [102]. However, long-term exposure to high-dose nanomaterials may cause local inflammation or endothelial cell destruction, which finally accelerate tumor metastasis [106, 107]. This is also observed in the nanomaterials which have been widely demonstrated to have high biosafety [108, 109]. Alternatively, FDA-approved drugs such as clodronate [33, 42], and the host-derived aging RBC [34, 110] have been used to temporarily delete macrophage barrier to enhance the lymphoid organ-targeted delivery efficiency of nanovaccines and reduce the dosage. Among them, host-derived aging RBC has much higher biocompatibility and is able to specifically and temporally delete macrophages in liver and spleen to extend the half-lives of nanovaccines. For instance, stressed RBCs (sRBCs, a kind of aging RBC obtained by heating fresh RBC) efficiently diminished the level of liver KCs and splenic red pulp macrophages within 16 h, whose level gradually can returne to normal after 72 h [110]. Moreover, a low dose (1.25 mg kg^−1^) of allogeneic anti-erythrocyte antibodies also efficiently produced aging RBC in vivo to delete liver KCs and splenic red pulp macrophages, which increased the circulation of a range of short-circulating and long-circulating NP formulations by up to 32-fold [34]. These indicate that aging RBC may be an efficient to tool to enhance spleen-targeted delivery efficiency of nanovaccines. We anticipate that more cutting-edge vaccine delivery strategies based on this will be developed to enhance the cancer immunotherapy and accelerate the clinical translation of cancer therapeutic vaccines.

## Abbreviations

TAAs: Tumor-associated antigens
APCs: Antigen-presenting cells
LN: Lymph node
s.c.: Subcutaneous
i.m.: Intramuscular
i.m.: Intratumoral
i.v.: Intravenous
PLGA: Poly(lactic-co-glycolic acid)
NPs: Nanoparticles
tdLNs: Tumor-associated draining lymph nodes
RBC: Red blood cell
DCs: Dendritic cells
LDH: Layered double hydroxide
OVA: Ovalbumin
TAMs: Tumor-associated macrophages
SCS: Subcapsular sinus
LECs: Lymphatic endothelial cells
MZ: Marginal zone
KCs: Kupffer cells
